# Sexualité des jeunes en milieu scolaire dans la ville de Likasi en République Démocratique du Congo

**DOI:** 10.11604/pamj.2018.31.34.16088

**Published:** 2018-09-14

**Authors:** Roger Tshimanga Mukadi, Maurice Sumaili Mwana Ntambwe, Hermann Tamubango Kitoko, Joseph Malashi Mwisi, Fiston Ilunga Mbayo

**Affiliations:** 1 Institut Supérieur des Techniques Médicales de Likasi, République Démocratique du Congo; 2 Service de Gynécologie Obstétrique Hôpital de Référence DACO, République Démocratique du Congo; 3 Centre de Santé de Référence Nkolomoni, République Démocratique du Congo; 4 Centre Médical Georgia, République Démocratique du Congo; 5 Centre de Santé Disanga II Lwambo, République Démocratique du Congo; 6 Université de Malemba Nkulu, République Démocratique du Congo

**Keywords:** Sexualité, jeunes, milieu scolaire, Likasi, Sexuality, young people, school environment

## Abstract

**Introduction:**

Des nombreuses études sur la sexualité en Afrique subsaharienne, notent un accroissement de l'activité sexuelle des jeunes célibataires, cette sexualité est précoce, instable, multipartenaire, dépendant de l'environnement proche des jeunes et des membres de leurs ménages. Cette étude vise à déterminer la prévalence des jeunes sexuellement actifs et évaluer les facteurs influant sur le comportement sexuel.

**Méthodes:**

Il s'agit d'une étude d'observation descriptive transversale couvrant la période allant de Mai à Juin 2017. L'étude a porté sur 249 élèves de 5^éme^ et 6^éme^ secondaires de 4 écoles de Likasi (CS Kalunga, CS la Borne, College Tutazamie et Lycée Musofi). Les données ont été saisies et encodées avec le logiciel excel 2013.

**Résultats:**

Deux cent quarante neuf jeunes ont répondu à l'enquête dont 153 filles et 96 garçons, parmi lesquels 104 sont sexuellement actifs soit une prévalence de 42%. L'âge Moyen au premier rapport sexuel chez le garçon est de 16,7 ans et 17,2 ans chez la fille, 38% ont eu leurs premiers rapports sexuels entre 10 et 15 ans, 56% déclarent avoir eu plus d'un partenaire sexuel. Le media, l'internet, le niveau et milieu de vie sont identifiés comme facteurs qui influencent le comportement sexuel des jeunes.

**Conclusion:**

Selon l'ONUSIDA, le SIDA s'est juvenilisé, l'élimination de cette épidémie passe par la promotion d'une sexualité saine et responsable selon la stratégie ABC (Abstinence, Bonne Fidélité, Condom).

## Abstract (en)

**Introduction:**

Numerous studies on sexuality conducted in sub-Saharan Africa report an increase in sexual activity among single young people. Adolescent population begins sexual activity earlier, has unstable relationships and multiple partners according to the proximity to the members of their households. This study aims to determine the prevalence of sexually active youth and to evaluate the factors influencing sexual behavior.

**Methods:**

We conducted an observational descriptive cross-sectional study over the period May -June 2017. The study focused on 249 pupils in a 5th, 6th grade at 4 secondary schools in Likasi (CS Kalunga, CS Terminal, College Tutazamie and Lycée Musofi). Data were entered and encoded using the excel software 2013.

**Results:**

Two hundred and forty-nine young people answered the survey (153 girls and 96 boys), 104 of whom were sexually active, with a prevalence of 42%. The average age of first sexual intercourse was 16.7 years for the boys and 17.2 years for the girls; 38% had had their first sexual intercourse between 10 and 15 years, 56% reported having had more than one sexual partner. Media, Internet, the living environment and the standard of living were factors influencing sexual behavior of young people.

**Conclusion:**

According to the UNAIDS, AIDS affect increasingly younger subjects; the elimination of this epidemic requires the promotion of healthy and responsible sexual life according to the ABC strategy.

## Introduction

L'entrée dans la vie sexuelle active des adolescents et jeunes représente une étape importante mais aussi très difficile pour la plupart des jeunes et adolescents. Car il faut concilier d'une part les aspects positifs et prometteurs de la sexualité «découverte, amour, partage», d'autres part les aspects négatifs «crainte d'une grossesse, des maladies sexuellement transmissibles et du SIDA» [1]. D'où la nécessité du programme sur la santé sexuelle et réproductive des jeunes et adolescents. L'adolescence est caractérisée par une augmentation de contact avec le sexe opposé. Ainsi à l'éveil sexuel de la puberté s'ajoute les dimensions sexuelles amoureuses, pour certains c'est le début derelation allosexuelle [2]. Les conditions d'entrée dans la vie sexuelle diffèrent d'une région à une autre. Les facteurs influençant cette entrée sont notamment; les normes siociales, la foi et la spiritualité, la tradition, les médias, le prolongement généralisé des études mais aussi et surtout les avancées enregistrées dans le domaine de la contraception et pour certains pays la légalisation de l'avortement [2, 3]. Des nombreuses études se sont penchées sur le concept de fécondité des adolescentes et ont noté un accroissement de l'activité sexuelle des célibataires [4] et donc des grossesses avant le mariage [5]. Actuellement, la socialisation sexuelle des femmes reste toujours plus contrôlée que celle des hommes, et leurs premiers rapports sont à la fois plus souvent tardifs et parfois encore mal vécus [3]. En France, l'âge au premier rapport sexuel est quasiment le même dans les jeunes générations (17,2 ans pour les hommes et 17,6 pour les femmes). La sexualité est diversement justifiée, le cunnilingus et la fellation sont devenus des pratiques courantes dans les deux sexes [6]. En Afrique subsaharienne, la sexualité préconjugale est intense, précoce, instable, multipartenaire, diversement justifiée, dépendant des caractéristiques socio-économiques, socio-démographiques et socio- culturelles des jeunes et des membres de leurs ménages [5].

En RDC, la tranche d'âge de 10-24 ans représente 28% de la population, 22% des jeunes de 15 ans ont déjà eu de rapport sexuel. Ce taux atteint 91% à 25 ans [5] A Kinshasa, près de 7 jeunes sur 10 sont déjà sexuellement actifs à l'âge de 18 ans, l'âge médian au 1^er^ rapport sexuel est de 15,98 ans. 87,5% des jeunes filles comme des garçons ont eu volontairement leur 1^er^ rapport, c'est-à-dire, un rapport consentant, non gratifié dans 84% de cas. Il s'agit le plus souvent d'un rapport non protégé et non conforme à la stratégie ABC [7]. A Lubumbashi, l'étude de Tabihta *et al.* (2015) montre que la fréquence des adolescents sexuellement actifs avoisine 56,9%, et l'âge moyen au premier rapport sexuel est de 12 ± 2,75 ans [8]. Devant ces faits socialement et normalement dérangeant les thèmes de recherche sur la sexualité des jeunes sont devenus une priorité. Surtout que l'environement ( contexte socio economique defavorable, polygamie, foyer mono parental) ect. dans lequel nous évoluons devient de plus en plus permissif entraînant ainsi une augmentation de l'activité sexuelle parmi les jeunes celibataires. La question principale de cette recherche est celle de connaitre les facteurs qui influencent les comportements sexuels des jeunes dans notre milieu. Nous pensons, cependant, que les facteurs qui influenceraient les comportements sexuels des jeunes seraient entre autres: la pauvreté, les medias, l'age, la religion, l'internet et le milieu de vie. Notre étude poursuit plusieurs objectifs à savoir; déterminer la prévalence des jeunes sexuellement actifs, ressortir les caractères socio-démographiques, culturels, économiques associé aux comportements sexuels des jeunes, évaluer le niveau de connaissance des jeunes sur l'infection à VIH/ Sida et le taux d'utilisation de la contraception.

## Méthodes

**Type et période d'étude:** Il s'agit d'une étude d'observation descriptive transversale couvrant la période allant de Mai à Juin 2017.

**Cadre d'étude:** La présente étude a été réalisée a Likasi dans la province du Haut Katanga en RDC.

**Population:** L'étude a porté sur les élèves du degré terminal (5 et 6 éme secondaires) de quatre écoles de la ville de Likasi.( College Tutazamie, CS La Borne, Lycée Musofi, CS Kalunga).

**Critères d'inclusion:** Ont été inclus; tout élève du degré terminal au secondaire, présent à l'école au moment de l'enquête. Tout élève ayant Accepté de répondre à l'enquête après tirage.

**Critères d'exclusion:** Ont été exclus; tout élève du degré terminal au secondaire mais absent de la classe ou présent mais pas en bonne santé (présent mais malade par exemple). Tout élève du degré terminal au secondaire ayant Accepté de répondre à l'enquête après tirage mais l'ayant fait en partie. Tout éleve du dégré terminal au secondaire absent de l'école au moment de l'enquete. Tout éléve du degré terminal ayant refusé de repondre à l'enquete.

**Taille de l'échantillon et technique d'échantillonnage:** La taille de l'échantillon a été calculée sur base de la formule suivante:

$$n=\frac{\left(e^{2}\cdot p\cdot q\right)}{d^{2}}$$

Pour un échantillon minimum attendu de n + n/10 n: taille minimale pour l'obtention de résultats significatifs pour un événement et un niveau de risque fixé. e: Niveau de confiance (la valeur type du niveau de confiance de 95%) était de 1,96 p: Probabilité de réalisation de l'événement ou la prévalence de la pathologie q: Complément de p = 1 - p d: marge d'erreur ou le degré de précision (généralement fixé à 5% ou 0,05). Avec une prévalence estimée à 38,8% en 2013 selon Cheik au Mali, la taille calculée de l'échantillon était de 365 plus 10% de 365 soit 402 individus à enquêter. Compte tenu de contrainte opérationnelle 249 élèves ont été retenus comme taille de l'échantillon dans notre étude. La technique d'échantillonnage aléatoire simple nous a permis de former notre échantillon.

**Gestion et collecte des données:** Les enquêteurs ont été répartis dans les écoles retenues où ils ont pu récolter les données par la technique de face à face.

**Variables étudiées:** Les variables étudiés sont: l'âge, sexe (masculin et féminin), l'adresse ou commune de provenance (Likasi, Kikula, Panda, Shituru), le type d'union des parents, la religion, l'âge au premier rapport sexuel, le contact sexuel (jeunes ayant un petit ami (e) mais sans rapport sexuel), le type de rapport, le contexte, la connaissance sur le VIH et IST, l'utilisation de contraceptifs, la méthode contraceptive utilisée, le lieu de rencontre et le canal d'information sur la sexualité.

**Analyses statistiques:** Les données ont été encodées sur l'office excel 2013 et analysées grace au logiciel Epi Info 7.1 et les variables qualitatives ont été générées sous forme de fréquence absolue et relative.

**Limite:** Les limites de l'étude restent cependant liées aux biais de déclaration et de mémorisation.

**Considérations éthiques:** Sur le plan éthique nous avons eu le consentement des élèves via les directeurs des écoles, nos données ont été gérées dans l'anonymat.

## Résultats

**Comportement sexuel:** Le Tableau 1 montre que 104 jeunes (42%) ont déjà eu un rapport sexuel, alors que 35 (14%) ont déclaré avoir eu des contacts sexuel c à d Reconnaissent avoir un partenaire sexuel mais sans avoir le rapport sexuel.

**Tableau 1:** Répartition des jeunes selon le comportement sexuel

Comportement sexuel	Effectif	Pourcentage
Jeunes Sexuellement Actifs	104	42
Jeunes ayant un Contact Sexuel	35	14
Jeunes non Sexuellement Actifs	110	44
Total	249	100

**Caractéristiques socio- démographiques des jeunes sexuellement actifs:** Le Tableau 2 renseigne 87 jeunes soit 84,0% avaient un âge inférieur à 20 ans, 54 jeunes soit 52% étaient de sexe féminin avec un sexe ratio 1,8 en faveur des jeunes filles, 69 jeunes soit 66,0% étaient dans une famille où les parents sont en couple, 59 jeunes soit 56,7% avaient un parent qui faisait une profession privée et 59 jeunes soit 56,7% provenaient de la commune de Likasi.

**Tableau 2:** Répartition des jeunes selon les caractéristiques socio-démographiques

Paramètres	Effectif (n=104)	Pourcentage
**Age (Année)**		
15 - 17	25	24
18 - 20	62	60
˃ 21	17	16
**Sexe**		
Féminin	54	52
Masculin	50	48
**Type d’union de parent**		
Vie en couple	69	66
Divorcé (Vie avec Papa)	7	7
Divorcé (Vie avec Maman)	15	14
Vie chez les grands parents	9	9
Autres( collatéraux )	4	4
**Profession**		
Fonctionnaire	45	43,3
Privée	59	56,7
**Provenance**		
Kikula	30	28,8
Likasi	59	56,7
Panda	10	9,6
Shituru	5	4,8

**Caractéristiques liés à l'activité sexuelle:** Le Tableau 2 rapporte que dans 82% des cas chez les jeunes garçons contre 63 % chez les jeunes filles le rapport était souhaité, comme pour les jeunes garçons que pour les jeunes filles le contexte était la jouissance personnelle soit 50% vs 31%, ce Tableau 3 rapporte l'âge de premier rapport sexuel chez les jeunes garçons qui était dans 46,3% entre 10 et 15ans; chez les jeunes filles dans 58% entre 16 et 20 ans. En rapport avec le type de rapport. De ce même tableau, le rapport a été consommé avec un seul parténaire dans 40% de cas pour les jeunes garçons comme pour les jeunes filles. De même dans 40% des cas, les jeunes filles l'ont consommé avec plusieurs parténaires. Dans le deux sexe, la contraception étaient une option de choix dans 76% de cas chez les garçons et 54% des cas chez les jeunes filles. Il se dégage du Tableau 4 que 48 jeunes soit 72% des jeunes ont utilsé le condom comme méthode contraceptive, la maison du partenaire était le lieu de rencotre chez 49 jeunes soit 47% et l'internet était le canal principal d'information chez 35 jeunes soit 33,8%.

**Tableau 3:** Répartition des jeunes selon caractéristiques liés à l’activité sexuelle dans les 2 sexes

Paramètres	Garçons (N=50)	Filles (N=54)
**Age du 1^ier^ rapport sexuel**	**Effectif (%)**	**Effectif(%)**
10-15	25 (46,3)	15 (30)
16 – 20	24 (44,4)	29 (58)
˃ 20	5(9,3)	6 (12)
**Type de rapport**		
Souhaité	41 (82)	34 (63)
Viol	4 (8)	12 (22)
Rapport sous l’effet d’un addictif	5 (10)	8 (15)
**Contexte de rapport**		
Jouissance personnelle	25 (50)	17 (31)
Pour faire plaisir au partenaire	23 (46)	15 (28)
Pour bénéficier les cadeaux	0 (0)	8 (15)
Non déterminé	2 (4)	14 (26)
**Nombre des partenaires**		
Un	21 (42)	24 (44)
Deux	15 (30)	6 (12)
Plusieurs	14 (28)	24 (44)
**Contraception**		
Oui	37 (76)	29 (54)
Non	12 (24)	19 (35)
Non déterminé	1 (2)	6 (11)

**Tableau 4:** Répartitions des jeunes selon caractéristiques liés à l’activité sexuelle

Paramètres	Effectif	Pourcentage
**Contraception utilisée**	**N=66**	
Condom	48	72
Pilule	7	11
Calendrier	7	11
Autres méthodes	4	6
**Lieu de Rencontre**	**N=104**	
Maison du partenaire	49	47
Hôtel	16	15
Ecole	3	3
Stade	6	6
Autres ( Ami,Oncle,Tante)	30	29
**Canal d’information**	**N= 104**	
Entretien avec les parents	12	11,5
Réseaux sociaux	18	17,3
Télévision	27	25,9
Internet	35	33,8
Livres et autres supports pornographiques	21	20,2
Musiques	2	1,9

**Figure 1:**
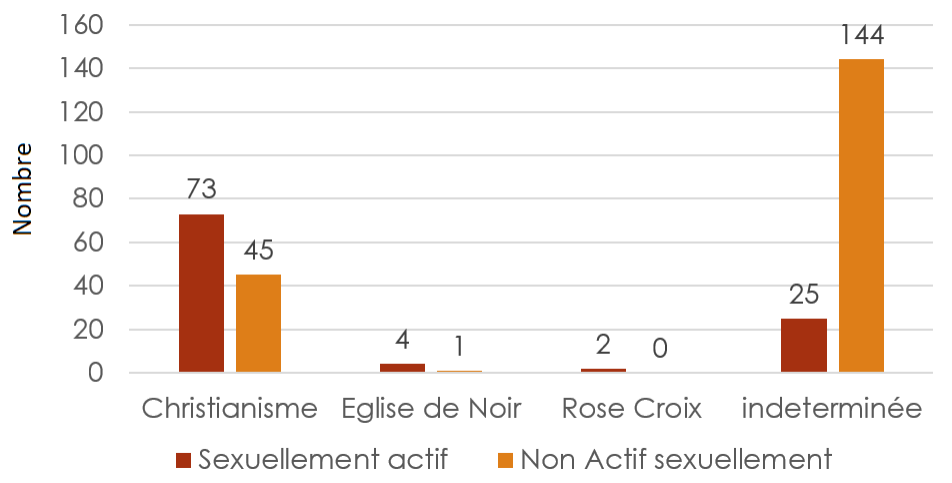
Répartition des jeunes sexuellements actifs selon leur croyance religieuse

**La croyance religieuse:** La figure rapportant sur la croyance religieuse montre que la majorité des jeunes sexuellement actifs croyaient au christianisme (Figure 1).

## Discussion

Dans notre série, la prévalence des jeunes sexuellement actifs est de 42% cette fréquence reste faible par rapport aux études ménées par Kambale (2015), Tabitha *et al.* (2015) et Ngongo (2015) qui ont raporté respectivement 95%, 56% et 52% [8-10]. Il est à noter que 14% entretenaient déjà les relations amoureuses. Nous osons croire que cette différence observée dans notre étude serait liée d'abord à la population cible puis à la taille de l' échantillon contrairement aux autres études non seulement nous avons porté notre intérêt sur les adolescents élèves mais aussi et surtaout ceux du dégré terminal (5ième et 6ième secondaire). Concernant le sexe, notre étude a rapporté un sexe ration de 1,8 en faveur du sexe feminin. Nous pensons que cela s'expliquerait du fait les jeunes filles entrent très tôt dans la vie génitale que les jeunes garçons de même âge. Par rapport à la provenance, 56% des jeunes dans notre étude sont venus de la commune de Likasi pour lesquels 66% desparents vivent en couple et 21% sont en divorce. Il a été constaté que les jeunes issus de milieu évolués ou bourgeois s'adonnent à la sexualité. L'explication serait l'accessiblité facile aux ressources internets pour lesquels les contenus privilégés sont les images et vidéo pornographiques. Il faudra dire que le milieu social joue un rôle non négligeable influant sur la pratique de l'activité sexuelle chez les jeunes adolescents. Ceci rejoint la théorie du capital social qui stipule que les comportements des individus se situent dans un contexte social qui renvoie au capital financier, humain et cadre social. Une étude menée à Kinshasa a montré que l'activité sexuelle devient un fait de mode et toute occasion pouvant permettre au jeune d'avoir des rapports sexuels ne peut qu'être exploitée. Lorsqu'on est objet des moqueries, on cherche à prouver aux amis (pairs) qu'on n'est pas impuissant ou frigide et qu'on peut faire comme eux [11]. La majorité des parents des jeunes sexuellement actifs vivent en couple, ceci met en cause l'idée selon laquelle, le foyer monoparental favoriserait une démission si pas un affaiblissement de suivi des parents dans l'éducation des jeunes.

Différentes études menées en Afrique et ailleurs témoignent que les normes sociales et culturelles imposées par la religion, la tradition et/ou la société ont d'importants effets sur la santé et le droit à la santé des jeunes [12]. Ainsi, La foi et la spiritualité servent de cadre éthique et influencent les croyances, les pratiques et les choix des jeunes par rapport à la famille, au mariage, à la procréation et à la sexualité. Dans notre série, 118 appartiennent au christianisme parmi lesquels 78 sont sexuellement actifs. Ceci qui contraste avec les enseignements du christianisme qui prônent la continence et la chasteté. 75% d'enquentés de l'eglise de noir sont sexuellement actifs. La religion semble ne plus influencée les comportements des jeunes sur la sexualité dans notre milieu. Kalambay *et al.* (2006) trouvent que 70% des jeunes sexuellement actifs appartiennent aux églises traditionnelles (Catholique, protestante et Kimbanguiste), alors qu'on pouvait s'attendre à une grande pratique de la continence et de la chasteté car ces derniers dominent souvent les enseignements dans ces églises [11]. Dans 60% des cas, nos enquetés ont l'âge compris entre 18-20 ans, l'âge moyen est de 18 ans, avec comme âge extrême 15 ans et 22 ans. L'âge Moyen au premier rapport sexuel chez le garçon est de 16,7 ans avec comme extrême 10 et 22 ans, tandis que chez la fille, l'âge moyen au 1^er^ rapport sexuel est de 17,2 ans avec comme extrême 10 et 23 ans. En Afrique subsaharienne, l'âge médian de la sexualité varie de 15 ans (Niger); 17,8 ans (Zimbabwe); 17,4 ans (Kenya); 18,8 ans (Sénégal) chez les femmes Selon le rapport de ONU/SIDA de 2010 [12]. En outre, la portion d'enquêtées qui ont reconnu avoir eu le premier rapport sexuel entre 10 et 15 ans, (40 Jeunes, soit 38%), est faible par rapport à l'étude de Goma (78%) pour la tranche d'âge de 10 à 14 ans, et celle de Lodja (49%). Cette proportion bien que faible par rapport à l' études de Ngongo *et al.* [10] montre une tendance vers la precocité sexuelle dans son milieu. Par ailleurs, plus la sexualité est précoce, plus le danger de connaitre les partenaires multiples s'accroit et expose ainsi les jeunes aux VIH, surtout que ce dernier s'est juvenilisé. Il s'agit le plus souvent d'un rapport souhaité, consenti 82% chez les garcons et 63% chez les jeunes filles, ces resultats corroborent avec les resultats de Kalambay *et al.* (2006) ou près de 87,5% des jeunes filles comme des garçons ont eu volontairement (après consentement) leur premier rapport sexuel. Pour certains jeunes 15% de cas de viol déclarés dans l'ensemble (absence du consentement du partenaire), cependant le cas des rapports sous l'effet de l'alcool et autres substances additives pouvait être assimilé au viol, mais relativisé du fait qu'il y a une difficulté de mettre en évidence l'état où le partenaire était dans l'impossibilité de donner un consentement [11]. Comme dans la serie de Kalambayi *et al.* (2006), les motifs du rapport sexuel sont principalement sentimentaux et non commerciaux, contrairement aux autres études sur la sexualité [9, 10] qui evoquent le monnayage de l'acte sexuel. Les filles s'adonnent à la sexualité contre une gratification financière ou des cadeaux qui leur permettent de satisfaire leurs besoins matériels et financiers. L'activité sexuelle devenant ainsi un moyen de survie et de se mettre à l'abri des besoins.

Les jeunes vivent une vie sexuelle intense, multipartenaire qui les expose certainement à de grands risques d'IST et du VIH, notre étude a montré que 56 jeunes (filles comme garçons) sur 104 (56%) déclarent avoir eu plus d'un partenaire sexuel,le nombre des partenaires sexuels est l'un des indicateurs incontournables du niveau de risque dans les pratiques sexuelles, plusieurs études démontrent que la sexualité des jeunes est multipartenaire [9-11] et donc à risque surtout pendant cette période où on assiste de plus en plus à une culture de *«friendswithbenefits»* c'est-à-dire les partenaires sexuels sans engagement amoureux avec comme conséquence des rapports sexuels occasionnels à l'extérieur du cadre amoureux traditionnel. Il ressort de nos analyses que le taux d'utilisations des condoms dans notre sérieest 74% dans l'ensemble, cependant, le condométait utilisé comme contraceptif afin d'éviter une grossesse non désirée et non dans la stratégie ABC pour la prévention de la transmission du VIH, Ngongo *et al.* rapporte un taux d'utilisation des préservatifstrès faible [10], Mais dans l'ensemble, le taux d'utilisation de la contraception atteint (63%), Guiella *et al.* (2006) trouvent que Parmi les jeunes qui ont déjà eu des rapports sexuels, 42% des filles et près d'un garçon sur deux a déjà utilisé une méthode contraceptive au cours de leur vie. La proportion de garçons de 15-19 ans qui a déjà utilisé une méthode contraceptive (52%) est au moins deux fois plus élevée que la proportion de garçons de 12-14 ans (23%) ayant déjà utilisé une méthode contraceptive [6]. Au Cameroun l'utilisation de la contraception est très faible parmi les jeunes sexuellement actifs [13]. La télévision est la principale source d'information sur la sexualité selon les études de François WAFO en 2012 (76,8%) alors que l'internet représenteprès de 59% selon la même étude, contre 65,5% pour l'ecole. dans 30% des cas les garcons seuls discutent de la sexualité avec leurs parents, contre 60% pour les filles. En effet, dans notre série, Les principaux canaux d'information sur la sexualité sont respectivement, l'internet (33,8%), la Télevison (25,9%), les livres et autres support pornographique 20%, ces resultats temoignent de l'influence des medias et des nouvelles techniques de l'information et de la communication sur les comportements sexuels des jeunes, nos resultats corroborent avec ceux de Djiré *et al.* (1997) qui montrent quel'information sur les dangers liés à la sexualité et les moyens de s'en protéger est pour la plupart du temps insuffisante [14]. Elle se transmet en dehors du cadre familial, souvent à travers les moyens modernes de communication. La majorité des enquetés ont des informations sur le VIH, plusieurs auteurs trouvent egalement que la majorité d'enquetés connaissent les moyens de transmission [9,10, 13] mais cette information ne semblepas influencée les comportements des jeunes dans le sens de l'abstinence. Contrairement aux resultats de Kalambay *et al.* qui rapporteque la majorité d'enquetés utilisaient le service hotelier [11], notre série montre que les enquetés se rencotrent dans la maison du partenaire (47%) et dans près de 29% chez un proche (amis, cousin, grand parent).

## Conclusion

Au terme de notre étude, la prevalence des jeunes sexuellement actifs en milieu scolaire dans la ville de likasi est importante, concernant plus le fille, bien qu'ayant des connaissances sur le VIH et IST les jeunes vivent une sexualité intense, multiparternaire qui les exposent au VIH et IST, au grossesse non desirée et donc à l'avortement non securisé, le plus souvent le motif du rapport sexuel est sentimemental. Le media, l'internet, le niveau et milieu de vie étaent les facteurs indentifiés comme influants sur les comportements des jeunes. Les objectifs de developpement durable qui visent audacieusement à enrayér le VIH SIDA d'ici 2030 ne peuvent être atteint que par la promotion d'une sexualité saine et résponsable selon la strategie ABC.

### Etat des connaissances actuelles sur le sujet

- Etudes de prévalences sur la sexualité chez jeunes en générales.

### Contribution de notre étude à la connaissance

- Ressortir le prévalence de jeunes sexuellement actifs dans un groupe d'élève du dégré terminal;
- Ressortir les différents facteurs associés à ce comportement sexuel dans ce groupe particulier qui est à la porte l'entrée dans la vie universitaire.

## Conflits d’intérêts

Les auteurs ne déclarent aucun conflit d'intérêts.

## Contributions des auteurs

Tous les auteurs ont contribué à la réalisation de ce travail; ils ont lu et approuvé la version finale du manuscrit.

## Remerciements

Nous remercions très sincérèrement les directeurs et les préfêts qui ont accepté que cette enquête puisse être fait de leurs institutions.
